# Supplementary material for: Genetic Characterization and Pathogenicity of H7N7 and H7N9 Avian Influenza Viruses Isolated from South Korea
Source: Viruses. 2021 Oct 13;13(10):2057. doi: 10.3390/v13102057 (PMC8540337; doi:10.3390/v13102057)
Supplement: Supplementary file 1 [file viruses-13-02057-s001.zip › viruses-1415344-supplementary.pdf]

Supplementary Figure S1. Phylogenetic tree of the NA9 (a) and NA7 (b) gene based on nucleotides. The tree was analyzed using the maximum-likelihood (ML) method with 1,000 bootstrap replication and only bootstrap values more than 50% are shown. The two H7 Korean avian influenza viruses (AIVs) isolated in this study are shown in bold. The human influenza viruses are indicated by red circles.

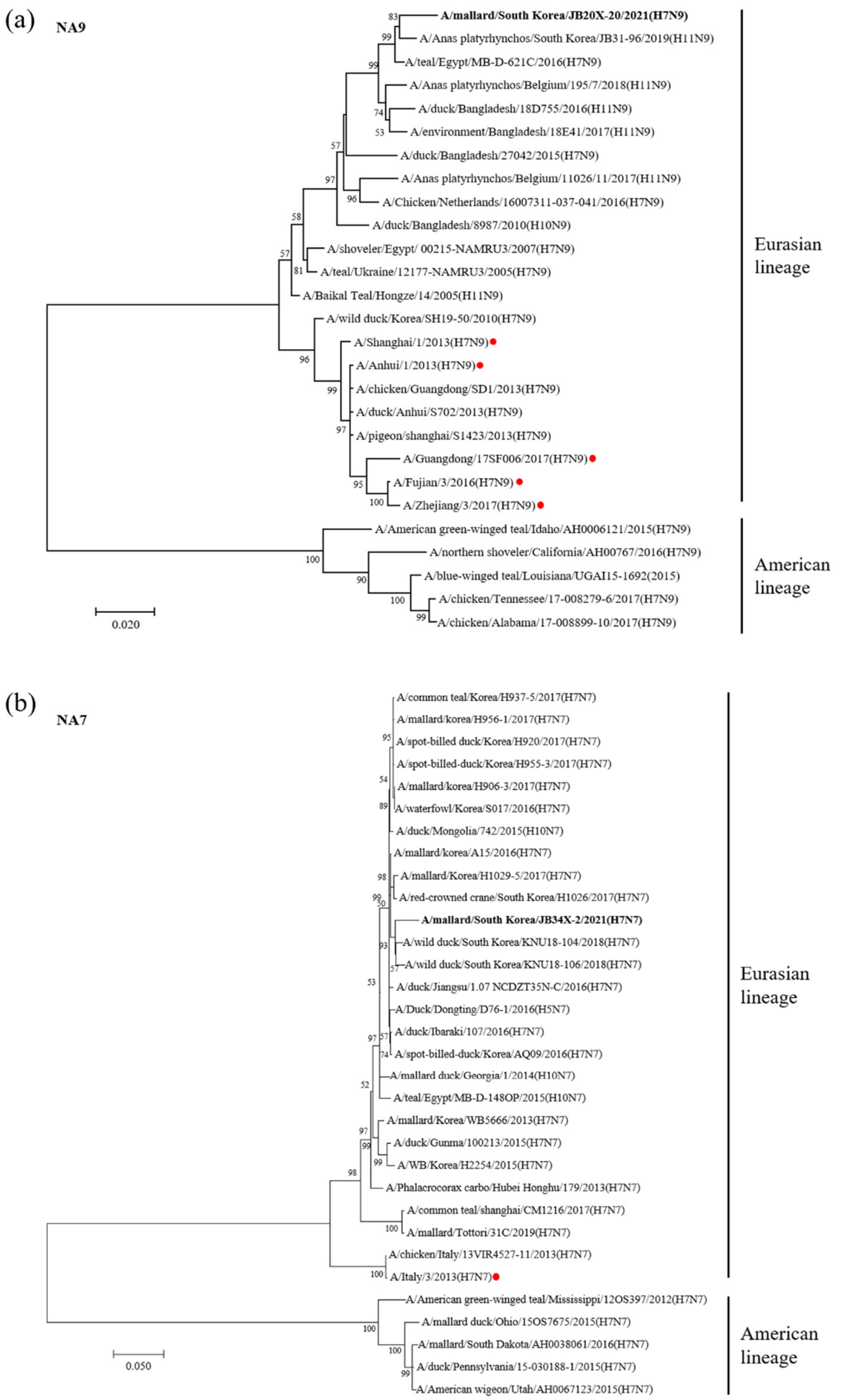

Supplementary Figure S2. Phylogenetic tree of the PB2 (a), PB1 (b), PA (c), NP (d), M (e), and NS (f) genes based on nucleotides. The tree was analyzed using the maximum-likelihood (ML) method with 1,000 bootstrap replication and only bootstrap values more than 50% are shown. The two H7 Korean AIVs isolated in this study are shown in bold. The human influenza viruses are indicated by red circles.

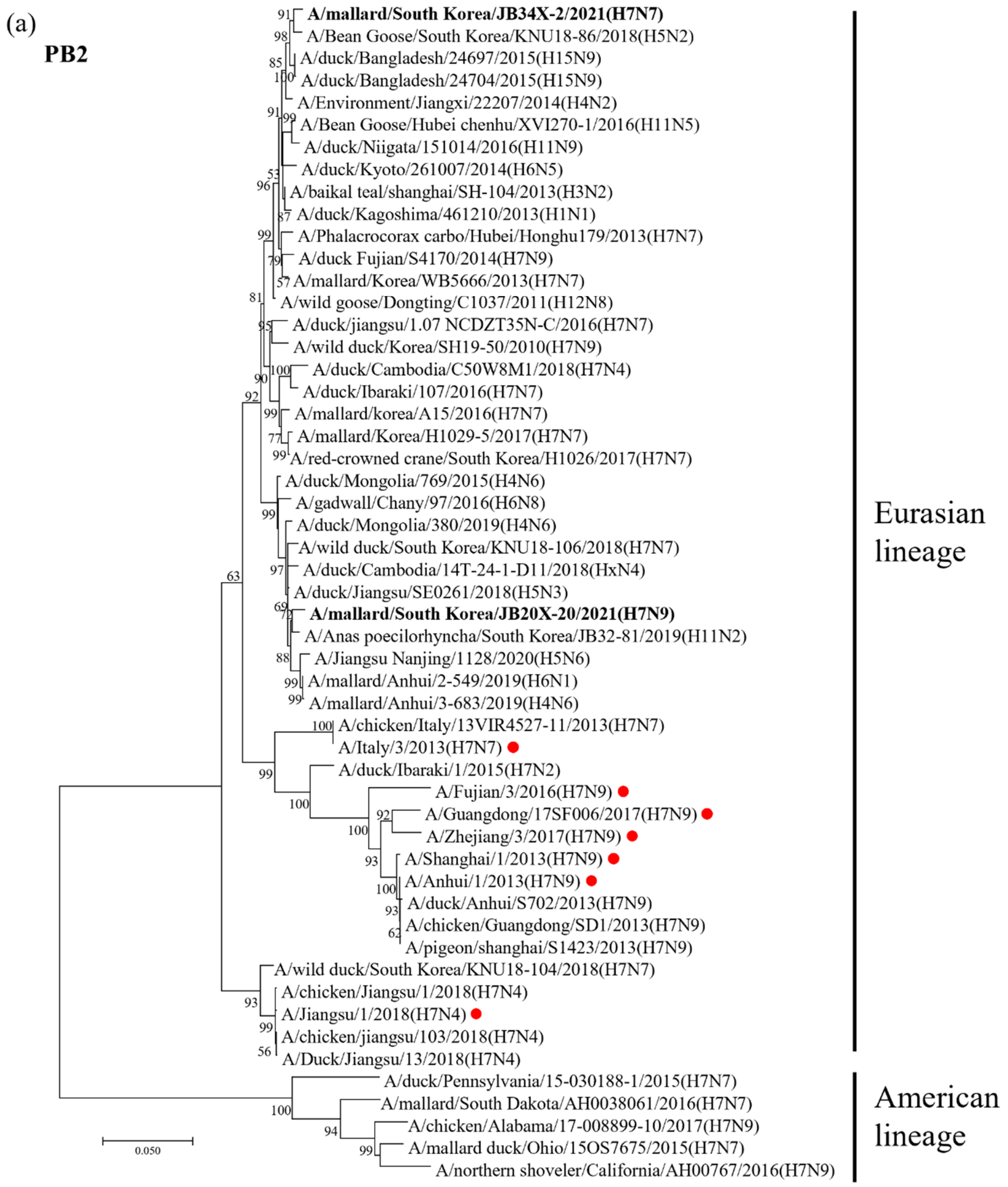

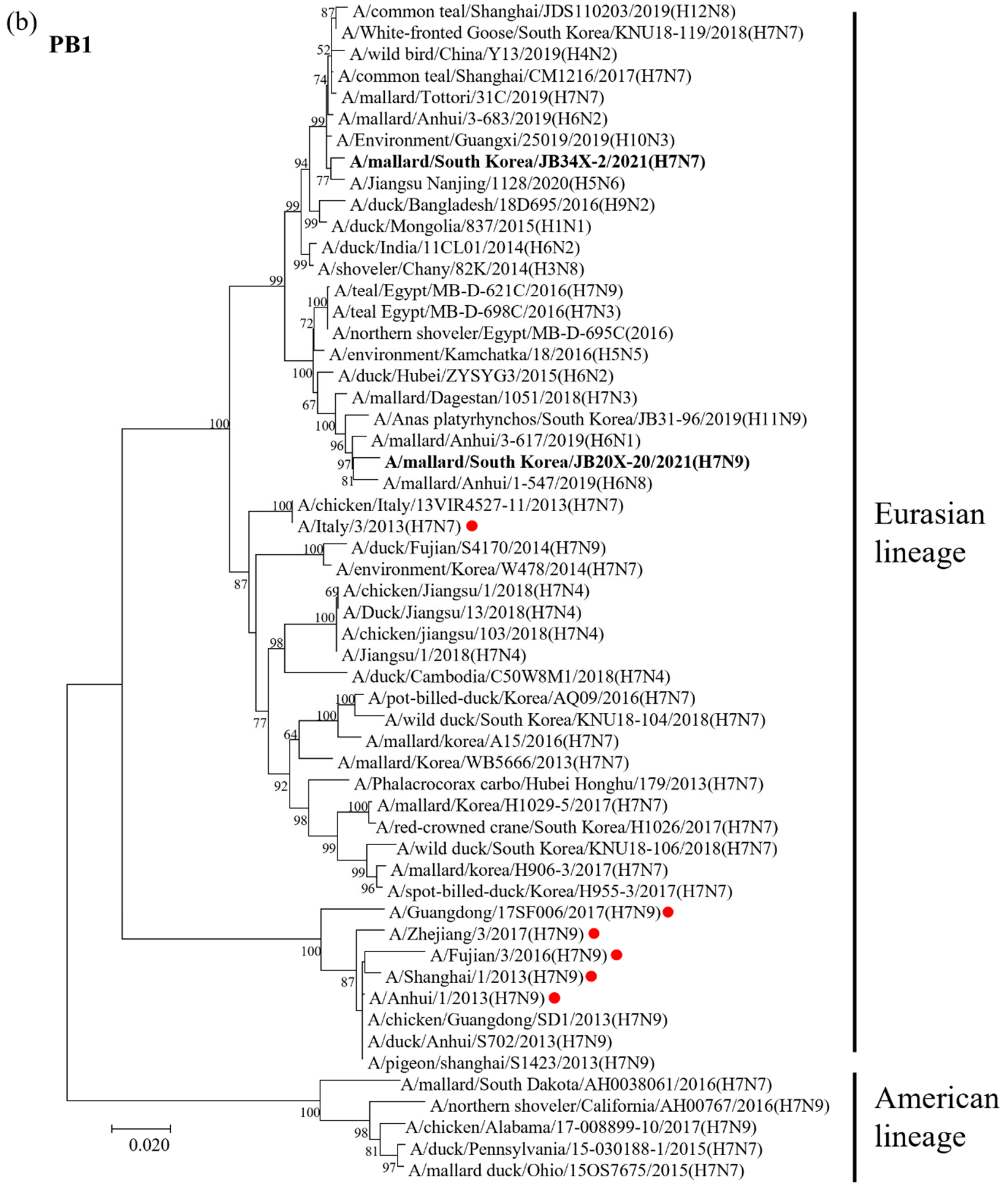
(b)
PB1
A/common teal/Shanghai/JDS110203/2019(H12N8)
A/White-fronted Goose/South Korea/KNU18-119/2018(H7N7)
A/wild bird/China/Y13/2019(H4N2)
A/common teal/Shanghai/CM1216/2017(H7N7)
A/mallard/Tottori/31C/2019(H7N7)
A/mallard/Anhui/3-683/2019(H6N2)
A/Environment/Guangxi/25019/2019(H10N3)
A/mallard/South Korea/JB34X-2/2021(H7N7)
A/Jiangsu Nanjing/1128/2020(H5N6)
A/duck/Bangladesh/18D695/2016(H9N2)
A/duck/Mongolia/837/2015(H1N1)
A/duck/India/11CL01/2014(H6N2)
A/shoveler/Chany/82K/2014(H3N8)
A/teal/Egypt/MB-D-621C/2016(H7N9)
A/teal Egypt/MB-D-698C/2016(H7N3)
A/northern shoveler/Egypt/MB-D-695C(2016)
A/environment/Kamchatka/18/2016(H5N5)
A/duck/Hubei/ZYSYG3/2015(H6N2)
A/mallard/Dagestan/1051/2018(H7N3)
A/Anas platyrhynchos/South Korea/JB31-96/2019(H11N9)
A/mallard/Anhui/3-617/2019(H6N1)
A/mallard/South Korea/JB20X-20/2021(H7N9)
A/mallard/Anhui/1-547/2019(H6N8)
A/chicken/Italy/13VIR4527-11/2013(H7N7)
A/Italy/3/2013(H7N7)
A/duck/Fujian/S4170/2014(H7N9)
A/environment/Korea/W478/2014(H7N7)
A/chicken/Jiangsu/1/2018(H7N4)
A/Duck/Jiangsu/13/2018(H7N4)
A/chicken/jiangsu/103/2018(H7N4)
A/Jiangsu/1/2018(H7N4)
A/duck/Cambodia/C50W8M1/2018(H7N4)
A/pot-billed-duck/Korea/AQ09/2016(H7N7)
A/wild duck/South Korea/KNU18-104/2018(H7N7)
A/mallard/korea/A15/2016(H7N7)
A/mallard/Korea/WB5666/2013(H7N7)
A/Phalacrocorax carbo/Hubei Honghu/179/2013(H7N7)
A/mallard/Korea/H1029-5/2017(H7N7)
A/red-crowned crane/South Korea/H1026/2017(H7N7)
A/wild duck/South Korea/KNU18-106/2018(H7N7)
A/mallard/korea/H906-3/2017(H7N7)
A/spot-billed-duck/Korea/H955-3/2017(H7N7)
A/Guangdong/17SF006/2017(H7N9)
A/Zhejiang/3/2017(H7N9)
A/Fujian/3/2016(H7N9)
A/Shanghai/1/2013(H7N9)
A/Anhui/1/2013(H7N9)
A/chicken/Guangdong/SD1/2013(H7N9)
A/duck/Anhui/S702/2013(H7N9)
A/pigeon/shanghai/S1423/2013(H7N9)
A/mallard/South Dakota/AH0038061/2016(H7N7)
A/northern shoveler/California/AH00767/2016(H7N9)
A/chicken/Alabama/17-008899-10/2017(H7N9)
A/duck/Pennsylvania/15-030188-1/2015(H7N7)
A/mallard duck/Ohio/15OS7675/2015(H7N7)
Eurasian lineage
American lineage
0.020

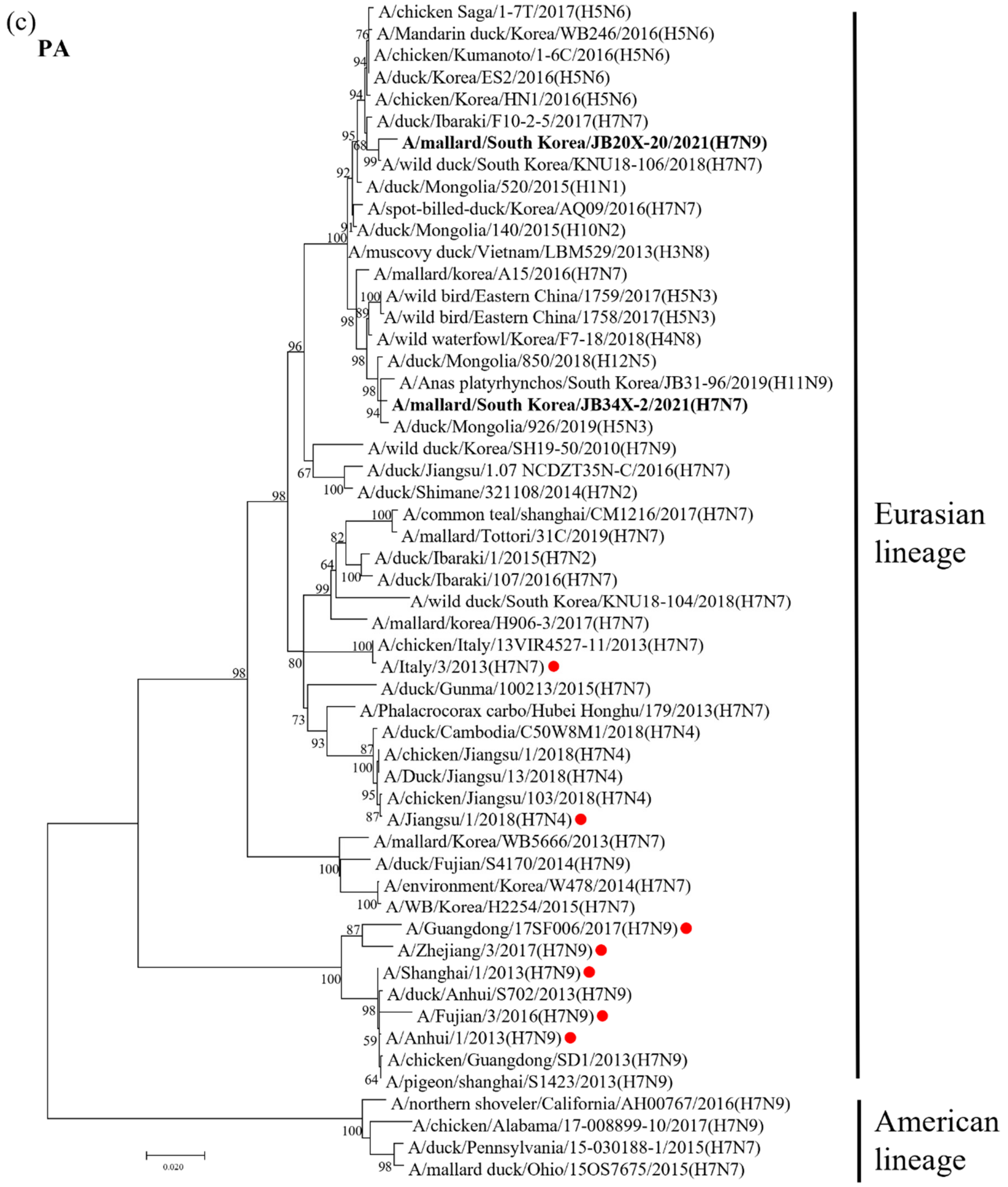
(c)
PA
A/chicken Saga/1-7T/2017(H5N6)
A/Mandarin duck/Korea/WB246/2016(H5N6)
A/chicken/Kumanoto/1-6C/2016(H5N6)
A/duck/Korea/ES2/2016(H5N6)
A/chicken/Korea/HN1/2016(H5N6)
A/duck/Ibaraki/F10-2-5/2017(H7N7)
A/mallard/South Korea/JB20X-20/2021(H7N9)
A/wild duck/South Korea/KNU18-106/2018(H7N7)
A/duck/Mongolia/520/2015(H1N1)
A/spot-billed-duck/Korea/AQ09/2016(H7N7)
A/duck/Mongolia/140/2015(H10N2)
A/muscovy duck/Vietnam/LBM529/2013(H3N8)
A/mallard/korea/A15/2016(H7N7)
A/wild bird/Eastern China/1759/2017(H5N3)
A/wild bird/Eastern China/1758/2017(H5N3)
A/wild waterfowl/Korea/F7-18/2018(H4N8)
A/duck/Mongolia/850/2018(H12N5)
A/Anas platyrhynchos/South Korea/JB31-96/2019(H11N9)
A/mallard/South Korea/JB34X-2/2021(H7N7)
A/duck/Mongolia/926/2019(H5N3)
A/wild duck/Korea/SH19-50/2010(H7N9)
A/duck/Jiangsu/1.07 NCDZT35N-C/2016(H7N7)
A/duck/Shimane/321108/2014(H7N2)
A/common teal/shanghai/CM1216/2017(H7N7)
A/mallard/Tottori/31C/2019(H7N7)
A/duck/Ibaraki/1/2015(H7N2)
A/duck/Ibaraki/107/2016(H7N7)
A/wild duck/South Korea/KNU18-104/2018(H7N7)
A/mallard/korea/H906-3/2017(H7N7)
A/chicken/Italy/13VIR4527-11/2013(H7N7)
A/Italy/3/2013(H7N7)
A/duck/Gunma/100213/2015(H7N7)
A/Phalacrocorax carbo/Hubei Honghu/179/2013(H7N7)
A/duck/Cambodia/C50W8M1/2018(H7N4)
A/chicken/Jiangsu/1/2018(H7N4)
A/Duck/Jiangsu/13/2018(H7N4)
A/chicken/Jiangsu/103/2018(H7N4)
A/Jiangsu/1/2018(H7N4)
A/mallard/Korea/WB5666/2013(H7N7)
A/duck/Fujian/S4170/2014(H7N9)
A/environment/Korea/W478/2014(H7N7)
A/WB/Korea/H2254/2015(H7N7)
A/Guangdong/17SF006/2017(H7N9)
A/Zhejiang/3/2017(H7N9)
A/Shanghai/1/2013(H7N9)
A/duck/Anhui/S702/2013(H7N9)
A/Fujian/3/2016(H7N9)
A/Anhui/1/2013(H7N9)
A/chicken/Guangdong/SD1/2013(H7N9)
A/pigeon/shanghai/S1423/2013(H7N9)
A/northern shoveler/California/AH00767/2016(H7N9)
A/chicken/Alabama/17-008899-10/2017(H7N9)
A/duck/Pennsylvania/15-030188-1/2015(H7N7)
A/mallard duck/Ohio/15OS7675/2015(H7N7)
Eurasian lineage
American lineage
0.020

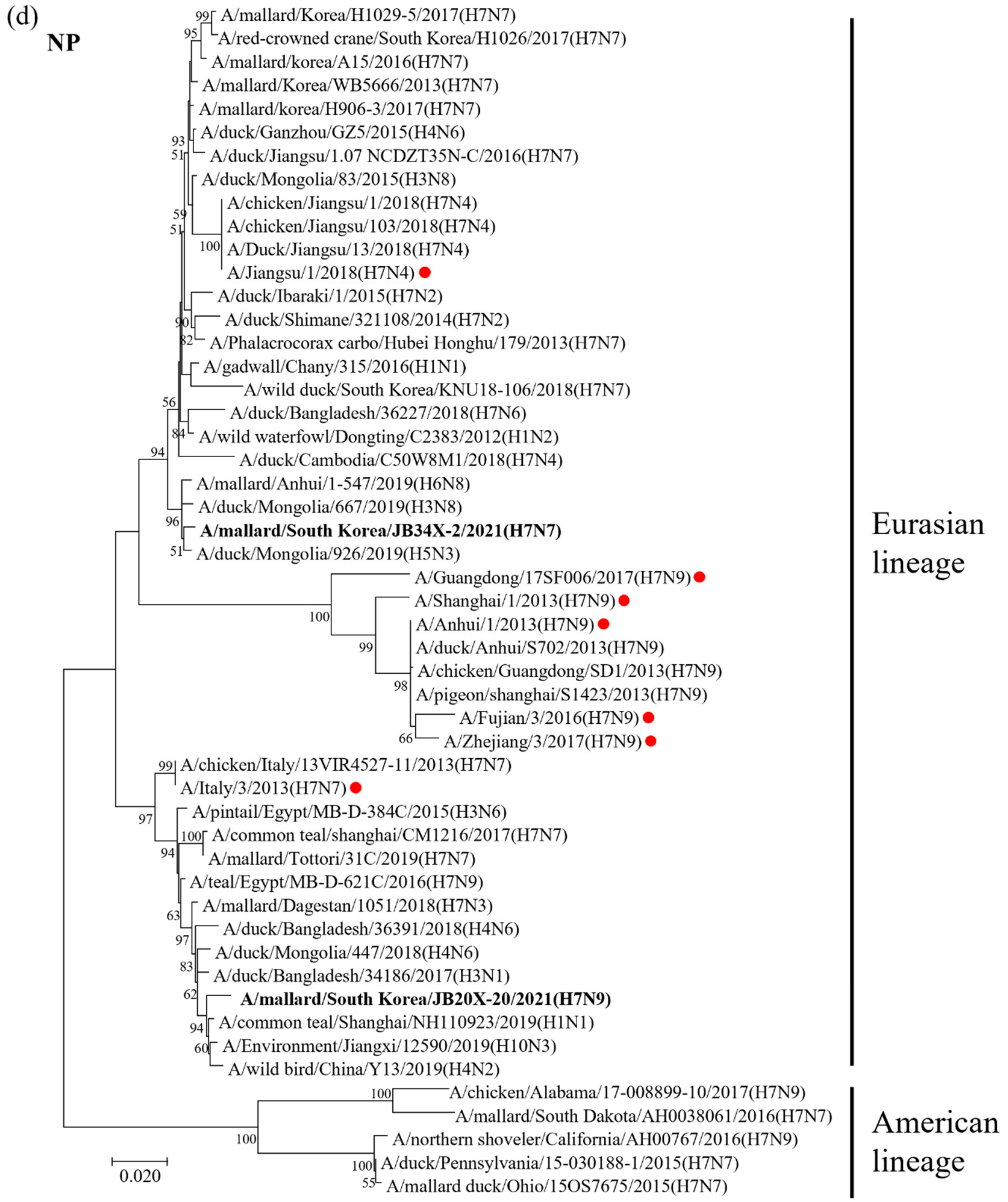
(d)
NP
A/mallard/Korea/H1029-5/2017(H7N7)
A/red-crowned crane/South Korea/H1026/2017(H7N7)
A/mallard/korea/A15/2016(H7N7)
A/mallard/Korea/WB5666/2013(H7N7)
A/mallard/korea/H906-3/2017(H7N7)
A/duck/Ganzhou/GZ5/2015(H4N6)
A/duck/Jiangsu/1.07 NCDZT35N-C/2016(H7N7)
A/duck/Mongolia/83/2015(H3N8)
A/chicken/Jiangsu/1/2018(H7N4)
A/chicken/Jiangsu/103/2018(H7N4)
A/Duck/Jiangsu/13/2018(H7N4)
A/Jiangsu/1/2018(H7N4)
A/duck/Ibaraki/1/2015(H7N2)
A/duck/Shimane/321108/2014(H7N2)
A/Phalacrocorax carbo/Hubei Honghu/179/2013(H7N7)
A/gadwall/Chany/315/2016(H1N1)
A/wild duck/South Korea/KNU18-106/2018(H7N7)
A/duck/Bangladesh/36227/2018(H7N6)
A/wild waterfowl/Dongting/C2383/2012(H1N2)
A/duck/Cambodia/C50W8M1/2018(H7N4)
A/mallard/Anhui/1-547/2019(H6N8)
A/duck/Mongolia/667/2019(H3N8)
A/mallard/South Korea/JB34X-2/2021(H7N7)
A/duck/Mongolia/926/2019(H5N3)
A/Guangdong/17SF006/2017(H7N9)
A/Shanghai/1/2013(H7N9)
A/Anhui/1/2013(H7N9)
A/duck/Anhui/S702/2013(H7N9)
A/chicken/Guangdong/SD1/2013(H7N9)
A/pigeon/shanghai/S1423/2013(H7N9)
A/Fujian/3/2016(H7N9)
A/Zhejiang/3/2017(H7N9)
A/chicken/Italy/13VIR4527-11/2013(H7N7)
A/Italy/3/2013(H7N7)
A/pintail/Egypt/MB-D-384C/2015(H3N6)
A/common teal/shanghai/CM1216/2017(H7N7)
A/mallard/Tottori/31C/2019(H7N7)
A/teal/Egypt/MB-D-621C/2016(H7N9)
A/mallard/Dagestan/1051/2018(H7N3)
A/duck/Bangladesh/36391/2018(H4N6)
A/duck/Mongolia/447/2018(H4N6)
A/duck/Bangladesh/34186/2017(H3N1)
A/mallard/South Korea/JB20X-20/2021(H7N9)
A/common teal/Shanghai/NH110923/2019(H1N1)
A/Environment/Jiangxi/12590/2019(H10N3)
A/wild bird/China/Y13/2019(H4N2)
A/chicken/Alabama/17-008899-10/2017(H7N9)
A/mallard/South Dakota/AH0038061/2016(H7N7)
A/northern shoveler/California/AH00767/2016(H7N9)
A/duck/Pennsylvania/15-030188-1/2015(H7N7)
A/mallard duck/Ohio/15OS7675/2015(H7N7)
Eurasian lineage
American lineage
0.020

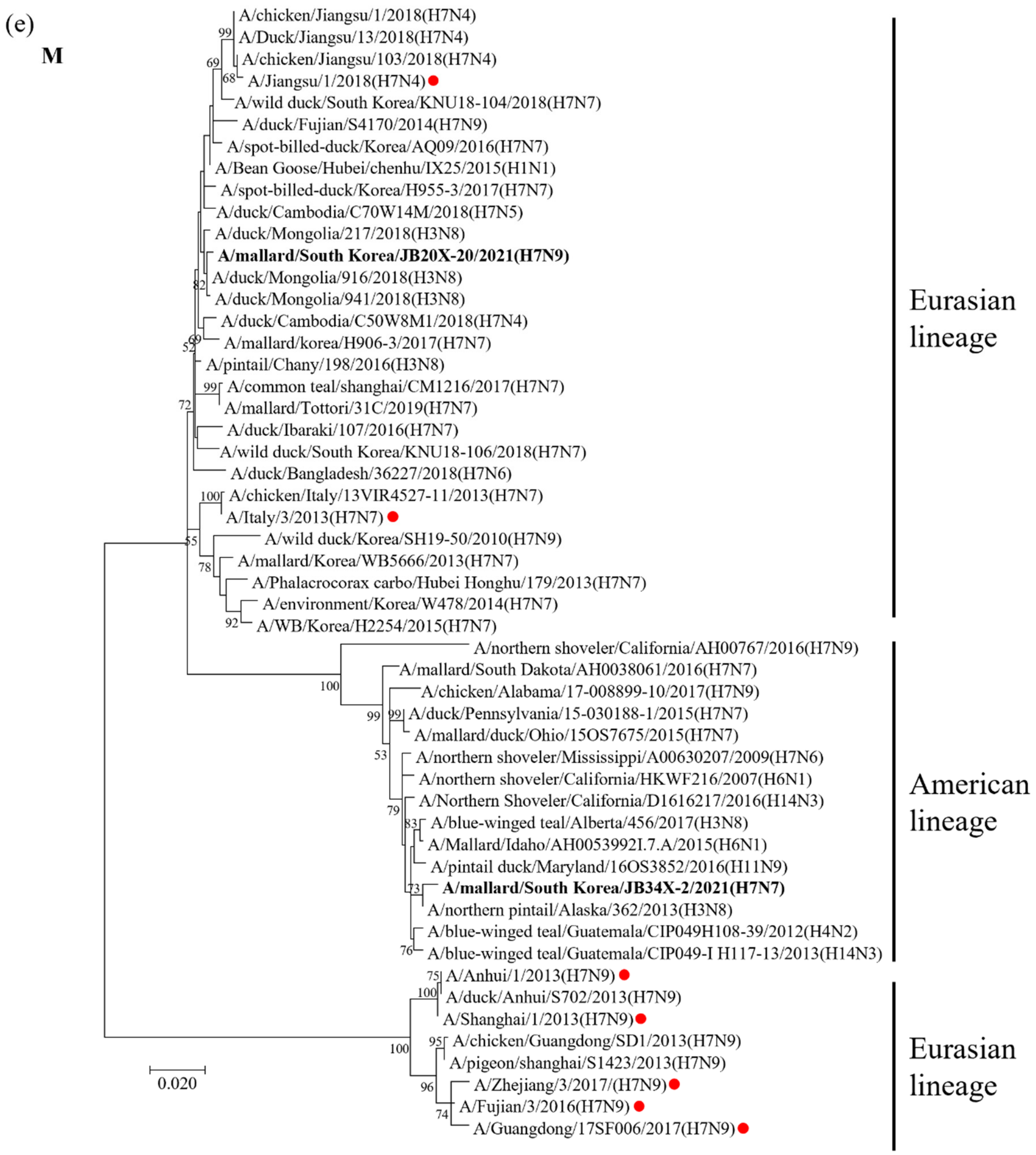
(e)
M
A/chicken/Jiangsu/1/2018(H7N4)
A/Duck/Jiangsu/13/2018(H7N4)
A/chicken/Jiangsu/103/2018(H7N4)
A/Jiangsu/1/2018(H7N4)
A/wild duck/South Korea/KNU18-104/2018(H7N7)
A/duck/Fujian/S4170/2014(H7N9)
A/spot-billed-duck/Korea/AQ09/2016(H7N7)
A/Bean Goose/Hubei/chenhu/IX25/2015(H1N1)
A/spot-billed-duck/Korea/H955-3/2017(H7N7)
A/duck/Cambodia/C70W14M/2018(H7N5)
A/duck/Mongolia/217/2018(H3N8)
A/mallard/South Korea/JB20X-20/2021(H7N9)
A/duck/Mongolia/916/2018(H3N8)
A/duck/Mongolia/941/2018(H3N8)
A/duck/Cambodia/C50W8M1/2018(H7N4)
A/mallard/korea/H906-3/2017(H7N7)
A/pintail/Chany/198/2016(H3N8)
A/common teal/shanghai/CM1216/2017(H7N7)
A/mallard/Tottori/31C/2019(H7N7)
A/duck/Ibaraki/107/2016(H7N7)
A/wild duck/South Korea/KNU18-106/2018(H7N7)
A/duck/Bangladesh/36227/2018(H7N6)
A/chicken/Italy/13VIR4527-11/2013(H7N7)
A/Italy/3/2013(H7N7)
A/wild duck/Korea/SH19-50/2010(H7N9)
A/mallard/Korea/WB5666/2013(H7N7)
A/Phalacrocorax carbo/Hubei Honghu/179/2013(H7N7)
A/environment/Korea/W478/2014(H7N7)
A/WB/Korea/H2254/2015(H7N7)
A/northern shoveler/California/AH00767/2016(H7N9)
A/mallard/South Dakota/AH0038061/2016(H7N7)
A/chicken/Alabama/17-008899-10/2017(H7N9)
A/duck/Pennsylvania/15-030188-1/2015(H7N7)
A/mallard/duck/Ohio/15OS7675/2015(H7N7)
A/northern shoveler/Mississippi/A00630207/2009(H7N6)
A/northern shoveler/California/HKWF216/2007(H6N1)
A/Northern Shoveler/California/D1616217/2016(H14N3)
A/blue-winged teal/Alberta/456/2017(H3N8)
A/Mallard/Idaho/AH00539921.7.A/2015(H6N1)
A/pintail duck/Maryland/16OS3852/2016(H11N9)
A/mallard/South Korea/JB34X-2/2021(H7N7)
A/northern pintail/Alaska/362/2013(H3N8)
A/blue-winged teal/Guatemala/CIP049H108-39/2012(H4N2)
A/blue-winged teal/Guatemala/CIP049-I H117-13/2013(H14N3)
A/Anhui/1/2013(H7N9)
A/duck/Anhui/S702/2013(H7N9)
A/Shanghai/1/2013(H7N9)
A/chicken/Guangdong/SD1/2013(H7N9)
A/pigeon/shanghai/S1423/2013(H7N9)
A/Zhejiang/3/2017/(H7N9)
A/Fujian/3/2016(H7N9)
A/Guangdong/17SF006/2017(H7N9)
0.020
Eurasian lineage
American lineage
Eurasian lineage

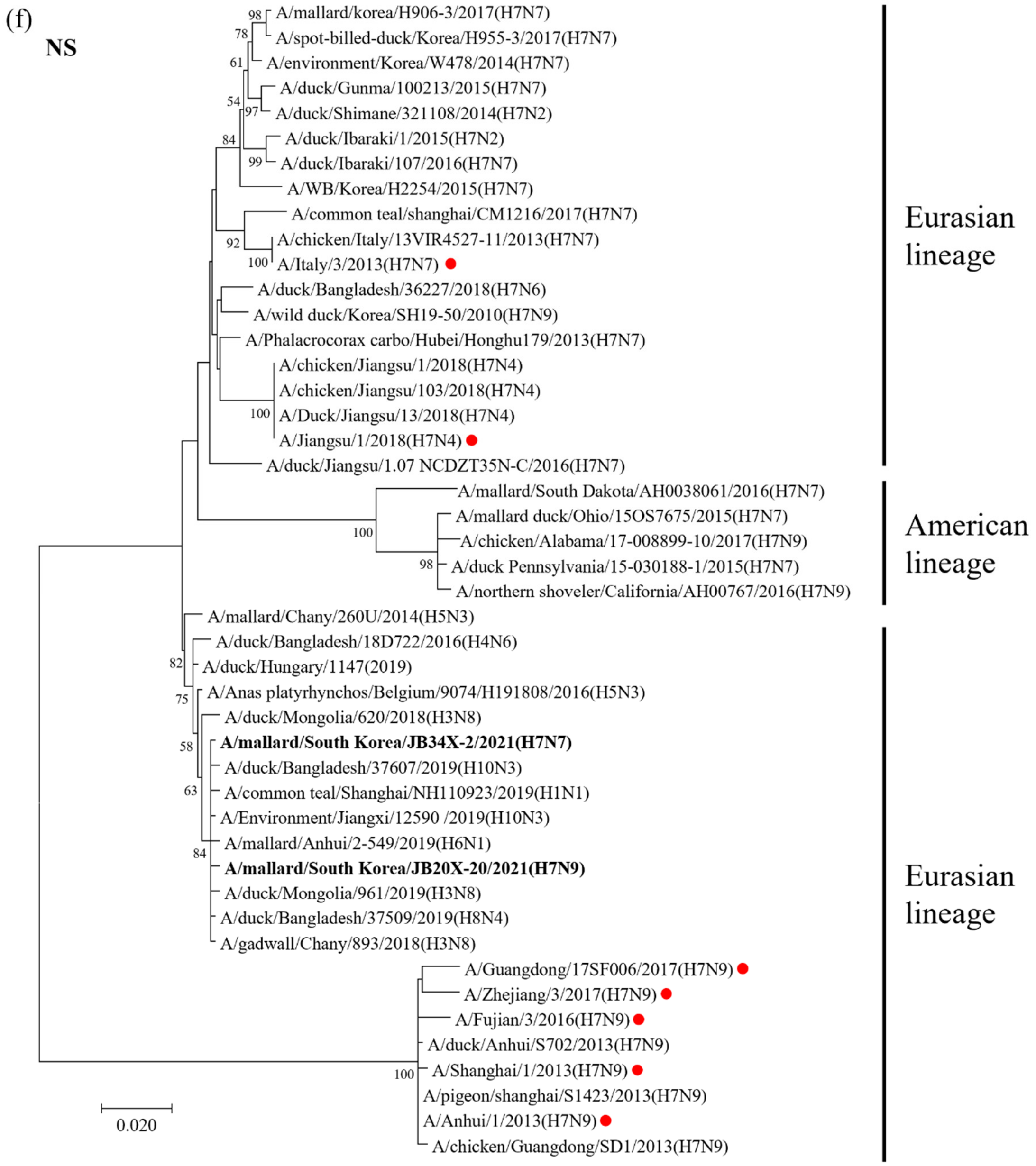
(f)
NS
A/mallard/korea/H906-3/2017(H7N7)
A/spot-billed-duck/Korea/H955-3/2017(H7N7)
A/environment/Korea/W478/2014(H7N7)
A/duck/Gunma/100213/2015(H7N7)
A/duck/Shimane/321108/2014(H7N2)
A/duck/Ibaraki/1/2015(H7N2)
A/duck/Ibaraki/107/2016(H7N7)
A/WB/Korea/H2254/2015(H7N7)
A/common teal/shanghai/CM1216/2017(H7N7)
A/chicken/Italy/13VIR4527-11/2013(H7N7)
A/Italy/3/2013(H7N7)
A/duck/Bangladesh/36227/2018(H7N6)
A/wild duck/Korea/SH19-50/2010(H7N9)
A/Phalacrocorax carbo/Hubei/Honghu179/2013(H7N7)
A/chicken/Jiangsu/1/2018(H7N4)
A/chicken/Jiangsu/103/2018(H7N4)
A/Duck/Jiangsu/13/2018(H7N4)
A/Jiangsu/1/2018(H7N4)
A/duck/Jiangsu/1.07 NCDZT35N-C/2016(H7N7)
A/mallard/South Dakota/AH0038061/2016(H7N7)
A/mallard duck/Ohio/15OS7675/2015(H7N7)
A/chicken/Alabama/17-008899-10/2017(H7N9)
A/duck Pennsylvania/15-030188-1/2015(H7N7)
A/northern shoveler/California/AH00767/2016(H7N9)
A/mallard/Chany/260U/2014(H5N3)
A/duck/Bangladesh/18D722/2016(H4N6)
A/duck/Hungary/1147(2019)
A/Anas platyrhynchos/Belgium/9074/H191808/2016(H5N3)
A/duck/Mongolia/620/2018(H3N8)
A/mallard/South Korea/JB34X-2/2021(H7N7)
A/duck/Bangladesh/37607/2019(H10N3)
A/common teal/Shanghai/NH110923/2019(H1N1)
A/Environment/Jiangxi/12590 /2019(H10N3)
A/mallard/Anhui/2-549/2019(H6N1)
A/mallard/South Korea/JB20X-20/2021(H7N9)
A/duck/Mongolia/961/2019(H3N8)
A/duck/Bangladesh/37509/2019(H8N4)
A/gadwall/Chany/893/2018(H3N8)
A/Guangdong/17SF006/2017(H7N9)
A/Zhejiang/3/2017(H7N9)
A/Fujian/3/2016(H7N9)
A/duck/Anhui/S702/2013(H7N9)
A/Shanghai/1/2013(H7N9)
A/pigeon/shanghai/S1423/2013(H7N9)
A/Anhui/1/2013(H7N9)
A/chicken/Guangdong/SD1/2013(H7N9)
0.020
Eurasian lineage
American lineage
Eurasian lineage
